# Harnessing the immunomodulatory effects of exercise to enhance the efficacy of monoclonal antibody therapies against B-cell haematological cancers: a narrative review

**DOI:** 10.3389/fonc.2023.1244090

**Published:** 2023-08-23

**Authors:** Harrison D. Collier-Bain, Frankie F. Brown, Adam J. Causer, Annabelle Emery, Rebecca Oliver, Sally Moore, James Murray, James E. Turner, John P. Campbell

**Affiliations:** ^1^ Department for Health, University of Bath, Bath, United Kingdom; ^2^ School of Applied Sciences, Edinburgh Napier University, Edinburgh, United Kingdom; ^3^ Department of Haematology, Royal United Hospitals Bath NHS Foundation Trust, Bath, United Kingdom; ^4^ School of Sport, Exercise and Rehabilitation Sciences, University of Birmingham, Birmingham, United Kingdom

**Keywords:** exercise, cancer therapy, natural killer cells, phagocytes, complement proteins, T-cells, B-cells

## Abstract

Therapeutic monoclonal antibodies (mAbs) are standard care for many B-cell haematological cancers. The modes of action for these mAbs include: induction of cancer cell lysis by activating Fcγ-receptors on innate immune cells; opsonising target cells for antibody-dependent cellular cytotoxicity or phagocytosis, and/or triggering the classical complement pathway; the simultaneous binding of cancer cells with T-cells to create an immune synapse and activate perforin-mediated T-cell cytotoxicity against cancer cells; blockade of immune checkpoints to facilitate T-cell cytotoxicity against immunogenic cancer cell clones; and direct delivery of cytotoxic agents via internalisation of mAbs by target cells. While treatment regimens comprising mAb therapy can lead to durable anti-cancer responses, disease relapse is common due to failure of mAb therapy to eradicate minimal residual disease. Factors that limit mAb efficacy include: suboptimal effector cell frequencies, overt immune exhaustion and/or immune anergy, and survival of diffusely spread tumour cells in different stromal niches. In this review, we discuss how immunomodulatory changes arising from exposure to structured bouts of acute exercise might improve mAb treatment efficacy by augmenting (i) antibody-dependent cellular cytotoxicity, (ii) antibody-dependent cellular phagocytosis, (iii) complement-dependent cytotoxicity, (iv) T-cell cytotoxicity, and (v) direct delivery of cytotoxic agents.

## Introduction

Monoclonal antibodies (mAbs) are standard care for many B-cell haematological cancers (discussed herein) (1, 2). The modes of action for these mAbs include: activating Fcγ-receptors (FcγR) on innate immune cells; opsonising target cells for lysis via cell-mediated cytotoxicity or phagocytosis, and/or initiating the classical complement pathway; the simultaneous binding of cancer cells with T-cells to create an immune synapse and activate perforin-mediated T-cell cytotoxicity (TCC); blockade of immune checkpoints to facilitate TCC; and direct delivery of cytotoxic agents following the internalisation of mAbs by target cells (3, 4). While mAb therapy – often combined with chemotherapy, radiotherapy and/or stem cell transplant – can result in a pathological complete response (5–12), inadequate responses and the persistence of minimal residual disease (MRD) increases the likelihood of treatment-resistant disease relapse among a subset of patients (13–16). Mechanisms of haematological cancer cell survival during mAb therapy are multifaceted but include: suboptimal immune effector cell frequency (17, 18); immune checkpoint overexpression (e.g., programmed cell death protein (PD)-1) (19); and the presence of regulatory proteins (e.g., CD47), and complement regulatory proteins (e.g., CD55, CD59, CD46) on the target cell surface which inhibit mAb mediated killing (20–22). In addition, the migration of haematological cancer cells across different lymphoid tissues (23) can promote their survival and proliferation in local niches (24).

Exercise might represent a non-pharmacological immunological adjuvant to mAb therapy, which could be harnessed to overcome mechanisms of treatment resistance. It is well established that a single (i.e., acute) bout of structured exercise – for example, aerobic exercise of 20- to 45-minutes duration – induces profound and transient changes to immune cell kinetics in humans, as reviewed elsewhere (25, 26). Indeed, as outlined next in Part 1 of this review, both immune cell frequency and overall functional competency have been shown to dramatically, and transiently change in blood and other tissues. This is primarily due to a leukocytosis in blood during exercise, and leukocytopenia in the hours after exercise cessation, which is thought to represent an egress of leukocytes to peripheral tissues. In Part 2, we outline how these immunomodulatory changes that arise from individual bouts of exercise might be harnessed to improve the treatment efficacy of mAbs – approved by the UK National Institute of Health and Care Excellence (NICE) (**Table 1**) – in B-cell haematological cancers. Specifically, we explain how single bouts of exercise might enhance mAb therapy efficacy by improving, (i) antibody-dependent cellular cytotoxicity, (ii) antibody-dependent cellular phagocytosis, (iii) complement-dependent cytotoxicity, (iv) T-cell cytotoxicity, and (v) direct delivery of cytotoxic agents. In doing so, we highlight that these exercise-induced changes may have the potential to improve mAb clinical responses and limit the persistence of MRD. Lastly, in Part 3 of this review, we summarise research areas where the immunomodulatory effects of a single bout of exercise might be explored in future as a means to augment the efficacy of mAb therapies against haematological cancers.

**Table 1:** Summary of monoclonal antibody immunotherapies recommended by the UK National Institute for Health and Care Excellence (NICE) and included in the British National Formulary (BNF) for the treatment of B-cell haematological cancers as of April 2023.

Drug	NICE^1^	Disease^1^	Isotype	Target	Effector function(s)
**Blinatumomab** **(Blincyto^®^)**	2017	Acute lymphoblastic leukaemia	Bispecific	CD19 CD3	Induces TCC by binding to CD3 in the T cell receptor complex and, subsequently, tethering CD19 on B cells (27). Blinatumomab is also associated with a transient upregulation of cell adhesion molecules, release of inflammatory cytokines and T cell proliferation (28).
**Brentuximab vedotin** **(Adcetris^®^)**	2018	Hodgkin lymphoma	IgG1	CD30	As antibody-drug conjugate that is internalised by CD30^+^ tumour cells, delivering conjugated monomethyl auristatin E that prevents tubulin polymerization, and results in cell cycle arrest and apoptosis (29).
**Daratumumab** **(Darzalex^®^)**	2019	Multiple myeloma	IgG1	CD38	Induces ADCC, ADCP and CDC against CD38^+^ tumour cells (30–32). Daratumumab also induces lysis of CD38^+^ MDSC, CD38^+^ T_regs_ and CD38^+^ B cells, and increases the absolute counts of CD8^+^ T cells (33).
**Inotuzumab ozogamicin** **(Besponsa^®^)**	2018	Acute lymphoblastic leukaemia	IgG4	CD22	An antibody-drug conjugate that is internalised by CD22^+^ tumour cells, delivering the conjugated anti-cancer antibiotic, N-acetyl-*γ*-calicheamicin, which induces double-stranded DNA breaks leading to cancer cell cycle arrest and apoptosis (34).
**Isatuximab** **(Sarclisa^®^)**	2020	Multiple myeloma	IgG*κ*	CD38	Induces ADCC, ADCP and CDC against CD38^+^ tumour cells (35). Similarly to daratumumab, isatuximab also induces lysis of CD38^+^ T_regs_, and increases the frequency of CD8^+^ T cells (36).
**Nivolumab** **(Opdivo^®^)**	2017	Classical Hodgkin lymphoma	IgG4	PD-1	Binds to PD-1 and, therefore, reduces PD-L1/PD-1 mediated immune suppression of T-cells (37).
**Obinutuzumab** **(Gazyvaro^®^, Gazyva^®^)**	2015 2018	Chronic lymphocytic leukaemia Follicular lymphoma	IgG1	CD20	Glycoengineered Fc-region that elicits enhanced ADCC and, to a lesser extent, ADCP in comparison to non-glycoengineered IgG1 antibodies. However, obintuzumab-induced CDC is impaired versus non-glycoengineered IgG1 antibodies (38).
**Pembrolizumab** **(Keytruda^®^)**	2018	Classical Hodgkin lymphoma	IgG4	PD-1	Binds to PD-1 and, therefore, reduces PD-L1/P-D1 mediated immune suppression of T-cells (39).
**Polatuzumab vedotin** **(Polivy^®^)**	2020	Diffuse Large B-cell lymphoma	IgG1	CD79B	An antibody-drug conjugate that is internalised by CD79B^+^ tumour cells, delivering conjugated monomethyl auristatin E, which prevents tubulin polymerization, and results in cell cycle arrest and apoptosis.
**Rituximab** **(Rituxan^®^, MabThera^®^)**	2008 2009 2012	Non-Hodgkin’s lymphoma Chronic lymphocytic leukaemia Follicular Lymphoma	IgG1	CD20	Induces ADCC, CDC and ADCP against CD20^+^ tumour cells (40).

^1^ Date of UK approval, and target diseases of the drugs extracted from the National Institute for Health and Care Excellence (41). ADCC, antibody dependent cellular cytotoxicity; ADCP, antibody dependent cellular phagocytosis; CDC, complement dependent cytotoxicity; TCC, T-cell cytotoxicity; MDSC, myeloid-derived suppressor cell; PD-1, programme cell death protein 1; PD-L1, programmed cell death ligand 1.

## Part 1: immune cell kinetics in response to a single bout of exercise

The effects of exercise on the immune system can be broadly categorised as: (i) acute – a transient response to a single bout (or session) of exercise; or (ii) adaptive – a cumulative (‘chronic’) effect of repeated exercise bouts. It is important to acknowledge that immunological adaptations to exercise training – for example, maintenance of T-cell repertoire and/or persistence – may augment the *direct* elimination of tumour cells, and indeed it is theorised that this is a key mechanism explaining why a physically active lifestyle lowers cancer risk and cancer mortality (26); this is strongly evidenced by human epidemiology studies showing that being physically active does not lower the risk of cancers characterised by a low mutational burden which may be immunologically ‘cold’ (26). Adding to the direct anti-cancer effects of regular exercise, we propose that the temporary immunological perturbations that arise in response to a single bout of exercise may be harnessed as an adjuvant that could also result in the elimination of haematological cancer cells, if undertaken alongside mAb therapy. Next, we discuss how single bouts of exercise affect effector immune cells that are instrumental in the cytotoxic effects of mAb therapy, and also how single bouts of exercise affect haematological cancer cells which may be susceptible to mAb-mediated killing.

**Figure 1:**
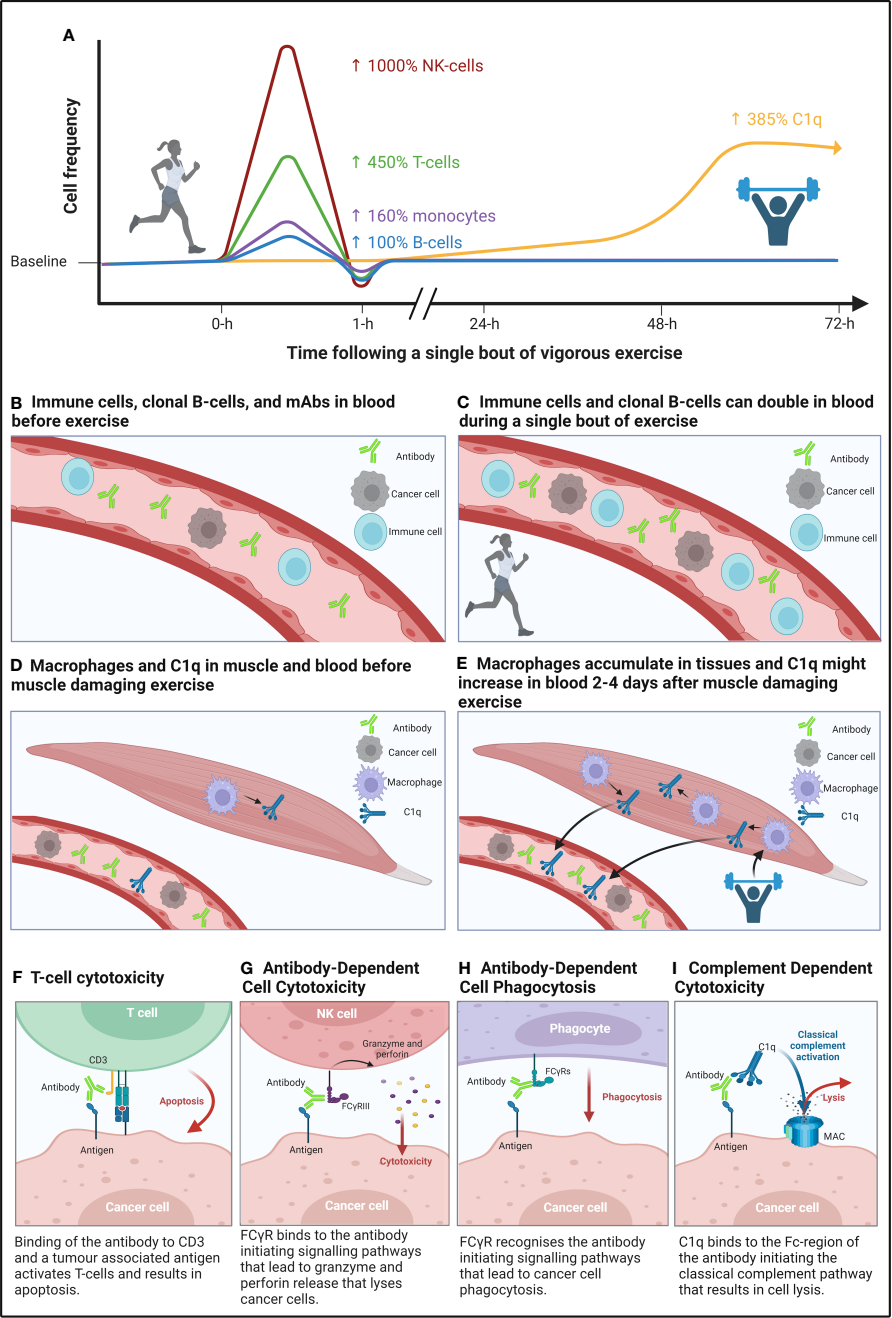
A graphical summary of the immunomodulatory effects of a single bout of aerobic, and muscle damaging eccentric exercise, and how these bouts might improve the mechanisms of action of monoclonal antibody (mAb) therapy. **(A)** Mobilisation of lymphocytes by high-intensity continuous exercise (left side of figure) is regulated by β2-adrenegic receptors, which preferentially mobilises cytotoxic NK- and T-cells, whilst also mobilising phagocytes (e.g., monocytes) and B-cells to a lesser extent, followed by an extravasation of these cells to peripheral tissues. Whereas complement system proteins of the classical pathway (e.g., C1q) may be increased two- to four-days following a bout of eccentric muscle damaging exercise (right side of figure). **(B, C)** Compares the frequency of immune cells and cancer cells in blood before and after a single bout of aerobic exercise. **(D, E)** Compares the frequency of macrophages – which secrete C1q – in muscle tissue following eccentric muscle damaging exercise and the purported ‘spill-over’ of C1q into blood. **(F-I)** Effector functions of mAbs that induce cancer cell death through binding to target cells to innate immune cells (e.g., NK-cells) and complexes (e.g., C1q). Figure created with BioRender.com.

It is well established that a single bout of moderate to vigorous intensity exercise mobilises immune cells into the blood of humans (**Figures 1A–C**). Whilst increased cardiac output and blood pressure result in a non-specific detachment of leukocytes from the vascular wall during exercise (42); stimulation of β2-adrenegic receptors preferentially mobilises lymphocytes capable of cytotoxic functions (43). Indeed, CD8^+^ T-cells (+25 to 450%) (44–52) and CD56^dim^ NK-cells (+88 to 982%) (44, 46, 53) are preferentially mobilised into blood during exercise, particularly at higher intensities (44, 46, 53). Furthermore, mobilised cytotoxic lymphocytes – immunophenotyped as CD158^+^NKG2A^−^ NK-cells and CD8^+^CD45RA^+^CCR7^−^ T-cells (44, 53) – have strong effector functions. For instance, an individual bout of cycling exercise augments NK-cell cytotoxicity against cancer cells *in vitro* (54). Beyond direct anti-tumour responses, the preferential mobilisation of highly cytotoxic lymphocytes is relevant to mAbs eliciting TCC and ADCC, which depend on T-cells and NK-cells, respectively, to elicit cancer cell killing, as discussed later in sections ‘T-cell cytotoxicity’ and ‘Antibody-dependent cellular cytotoxicity’ – in Part 2. Transient changes to the frequency of other immune cells in response to individual exercise bouts may also enhance mAb therapy. For example, transient exercise-induced mobilisation of monocytes (+100-480%) (55–57) into the circulation may enhance the efficacy of mAb therapies that act via ADCP (see ‘Antibody-dependent cellular phagocytosis’ – in Part 2). Additionally, reductions to T_reg_ frequency (−84%) from pre- to post-exercise (58) may alleviate T-cell anergy to potentiate the effects of mAbs that induce TCC, although it should be noted that T_reg_ frequency has also been shown to be increased or unchanged in response to individual exercise bouts, as reviewed elsewhere (59), as discussed later in section ‘T-cell cytotoxicity’ – in Part 2. Furthermore, given that B-cell lineage cancers comprise the majority of haematological cancers, it is relevant to consider that CD19^+^CD20^+^ B-cells increase (+0 to 99%) in blood in response to individual exercise bouts (44, 45, 48–50, 60, 61). An exercise-induced increase in B-cell frequency may have implications for mobilising clonal cancer cells from protective stromal niches – which could in turn facilitate binding of mAb to target cells when infused into the blood, and thus enhance elimination via ADCC, ADCP, CDC, TCC, or direct delivery of cytotoxic agents (see ‘Clonal B-cell mobilisation’ – in Part 2).

In addition to changes to cell frequency, complement system proteins – a compartment of the innate immune system capable of lysing cancer cells in the presence of mAb – are activated following individual sessions of exercise (62). Human studies have shown that complement proteins (including those of the C1-complex, e.g., C1s) are increased for up to three days following ultra-endurance and resistance exercise, respectively (63–65). In addition, rodent studies have shown that C1q secretion by M2-like macrophages in damaged skeletal muscle, peaks in serum two to four days post-injury (**Figures 1D, E**) (66, 67). The potency of mAb therapies that elicit cancer cell killing via CDC may therefore be enhanced by individual exercise bouts that induce skeletal muscle damage (see ‘Complement dependent cytotoxicity’ in Part 2).

## Part 2: evaluating how exercise induced immunomodulation can improve the efficacy of monoclonal antibody immunotherapy

In Part 2, we summarise the modes of action of mAb therapies that are used to treat haematological cancers, the mechanisms of resistance to those mAb therapies, and how individual bouts of exercise may overcome these mechanisms of resistance to improve the efficacy of mAb therapies in haematological cancers.

### Antibody-dependent cellular cytotoxicity

Antibody-dependent cellular cytotoxicity (ADCC) is a primary mechanism of several anti-cancer mAbs (**Table 1**). NK-cells, monocytes, macrophages, and granulocytes are capable of inducing ADCC (68, 69); however, most mAbs used to treat haematological cancers rely on NK-cells as the principal inducer of ADCC (70, 71) and, therefore, NK-cell mediated ADCC is discussed herein. ADCC is initiated upon mAb binding to molecules of a specific target antigen, whereby the Fc-region on IgG binds to FcγRIIIA/CD16a or FcγRIIC/CD32c on NK-cells (72, 73). mAb-bound NK-cells – primarily of CD56^dim^CD16^+^ immunophenotype – lyse target cells through the exocytosis of perforins and granzymes (**Figure 1G**), while IFN-γ secretion by NK-cells promotes an adaptive immune response (74).

Treatment of haematological cancers with mAbs often results in depletion of the total NK-cell count, including CD56^dim^CD16^+^ NK-cells, which are vital for mAb-mediated ADCC, and typically remain <30% of pre-treatment values throughout therapy (30, 75). This may be the result of mAb targets (e.g., CD38) being expressed by both target cells and effector cells (30, 76). Additionally, activation of NK-cells in response to target cells may induce CD16 shedding mediated by an activation of matrix metalloproteinases (77, 78) which could limit the ability for repeated ADCC activity by individual NK-cells. An individual bout of vigorous intensity exercise has been shown to cause a 10-fold increase in circulating CD56^dim^ NK-cells (44) and a preferential increase in the proportion of CD56^+^CD16^+^ NK-cells (45), leading to enhanced NK-cell cytotoxic potential (54). Therefore, it could be hypothesised that this exercise induced process may counter mAb-induced lymphopenia of effector cells and recruit NK-cells with functioning CD16 into blood. It is important to note that although increased NK-cell frequency may improve the effectiveness of mAb therapy, it may also result in a greater overall reduction in NK-cells. Therefore, a ‘trade-off’ between improved treatment efficacy and reduced NK-cell frequency should be considered in future research.

Immature CD56^bright^ NK-cells, which strongly produce IFN-γ but retain low cytotoxic activity, are characterised by strong expression of inhibitory receptors (e.g., NKG2A) (79). A loss of NKG2A, with a concomitant gain of killer-cell immunoglobulin-like receptors (KIR), is indicative of differentiation to cytotoxic CD56^dim^ NK-cells (79). In the context of exercise, serum collected 1-hour after a moderate to vigorous intensity bout of cycling exercise reduced the proportion of NK-cells with an inhibitory phenotype (NKG2A^+^NKG2C^−^) *in vitro*. These changes were associated with reduced cortisol and increased IFN-γ in serum, and resulted in enhanced lysis of multiple myeloma and lymphoma cell lines (80). Malignant B-cells commonly express human leukocyte antigen (HLA)-E and evade NK-cell cytotoxic activities through inhibitory NKG2A/HLA-E signalling. Thus, anti-NKG2A mAb enhance ADCC against HLA-E^+^ B-cells (81, 82) and exercise-induced downregulation of NKG2A may similarly augment ADCC. A downregulation of inhibitory-KIR has also been observed in the presence of pro-inflammatory cytokines such as IL-12 and IL-15 (83) – which are elevated in serum following exercise (84–86) – and may be a further means for individual bouts of exercise to augment ADCC through preventing NK-cell inhibitory signalling by haematological cancers (87).

Taken together, there is a clear rationale in haematological cancers to investigate exercise as an adjunct to mAbs that function through ADCC, yet, to date, studies that describe NK-cell mobilisation following individual exercise bouts have been undertaken in healthy people or patients with solid tumours. Thus, future research is required to determine NK-cell kinetics and NK-cell functionality in response to exercise in people with haematological cancers.

### Antibody-dependent cellular phagocytosis

Antibody-dependent cellular phagocytosis (ADCP) is a crucial contributor to the efficacy of many therapeutic mAbs used to treat haematological cancers (**Table 1**). Both macrophages and monocytes are capable of eliciting ADCP (88–90), however, macrophages are considered the predominant effector cell, due to their abundance in tumour microenvironments (91). Macrophages are typically tissue resident cells (92), and when found in solid tumours they are often referred to as tumour associated macrophages (TAMs). In haematological cancers, given the great diversity in the tumour landscape and thus microenvironment of different tumours, it is important to note that TAMs may be referred to as: leukaemia-associated macrophages (LAMs), acute leukaemia-associated macrophages (AAMs), and nurse-like cells (NLCs) (93). For the purpose of this review, these haematological cancer associated macrophages will be referred to as TAMs. ADCP is induced when FcγR – FcγRIIA/CD32a and FcγRIIIA/CD16a – on macrophages and monocytes bind to the Fc-region of target cell-bound mAb, resulting in the internalisation of the mAb and destruction of the target cell via phagosome acidification (88, 94) (**Figure 1H**).

In the blood, monocytes are likely to be the primary effector cell capable of eliciting ADCP (95). Monocyte-mediated ADCP can be impaired as a result of treatment-related downregulation of CD16 expression (95). However, individual bouts of vigorous intensity exercise, lasting 35-seconds to 45-minutes, have been shown to increase CD14^+^CD16^+^ monocytes by 100-480% in blood (55–57), which may temporarily overcome treatment-related CD16 downregulation and augment mAb-mediated ADCP. It is important to note that these studies recruited healthy people. In the context of haematological cancers, patients exhibit greater frequencies of circulating, immunosuppressive monocytic (M)-MDSCs (96, 97), which can be distinguished from healthy monocytes by low or no expression of the MHC class II molecule – HLA-DR (98). If regular exposure to individual bouts of exercise during mAb therapy can transiently increase the frequency of circulating HLA-DR^+^CD14^+^CD16^+^ monocytes in patients with haematological cancer, then ADCP could be enhanced.

CD14^+^CD16^+^ monocytes mobilised by individual exercise bouts may also express CX3CR1 and CXCR4 (99). Therefore, monocytes possess migratory potential to some tumour microenvironments – such as the bone marrow – where haematological cancer cells often reside and escape mAb-mediated killing (100) – via the CX3CL1/CX3CR1 (101, 102) and CXCR4/CXCL12 signalling axes (103). As such, it is plausible that an increased ratio of monocytes to cancer cells within the tumour microenvironment could result in augmented mAb-mediated ADCP (104). Monocytes may also differentiate into haematological TAMs in the tumour microenvironment (105, 106), which might be capable of individually phagocytosing multiple haematological cancer cells during an ADCP response (31). Thus, monocyte differentiation provides another mechanism by which exercise-induced monocyte mobilisation may bolster the depth of mAb-mediated ADCP.

Haematological TAMs are typically pro-oncogenic (M2-like), whilst M1-like macrophages are considered anti-oncogenic (106, 107) and thus, are capable of eliciting greater ADCP (105, 108, 109). Repeated bouts of aerobic exercise have the potential to manipulate TAM phenotypes in the tumour microenvironment. For instance, a 7,12-dimethylbenz(a)anthracene (DMBA) induced tumour mouse model showed tumour bearing mice forced to exercise exhibited M1-like TAMs, whereas control/inactive tumour bearing mice exhibited M2-like TAMs (110). However, the tumour microenvironment of haematological cancers is diverse; thus, understanding the polarization of TAMs in patient groups is challenging (107, 111). Future research should consider the differences in the haematological TAM location and where mAb-mediated ADCP takes place (e.g., blood, lymphoid tissues). Nonetheless, it might be hypothesised that repeated bouts of moderate to vigorous intensity exercise – over the course of mAb therapy – may re-educate macrophages towards an M1-like phenotype, thus improving mAb-mediated ADCP (112). However, ADCP is restricted by cancer cell expression of CD47, which interacts with signal regulatory protein-α (SIRP-α/CD172a) on the surface of macrophages, initiating an anti-phagocytic ‘*don’t eat me*’ signal (20). This evasion mechanism may limit the ability of exercise to enhance ADCP, as no studies have explored the effects of acute or regular exercise on haematological cancer cell CD47 expression.

Evidence of exercise-induced mobilisation and re-education of monocytes/macrophages is limited, and studies to date have recruited healthy people, or have examined solid tumour models in rodents. Nonetheless, the evidence summarised herein provides an encouraging rationale for future research to determine monocyte/macrophage kinetics, function, and phenotype in response to individual exercise bouts among people with haematological cancers.

### Complement-dependent cytotoxicity

Complement-dependent cytotoxicity (CDC) is a mode of action of many mAbs used to treat haematological cancers (**Table 1**). The binding of C1q with the Fc-region of target cell-bound mAb activates a proteolytic cascade of events resulting in the assembly of a membrane attack complex on the tumour cell surface, causing altered cell permeability and subsequent cell lysis (113) (**Figure 1I**). CDC efficacy in haematological cancers is limited by the overexpression of fluid phase (e.g., Factor H, C1-inhibitor, C4Bp), and membrane bound (e.g., CD55, CD59, CD46) complement regulatory proteins (21, 22), as well as reduced bioavailability of crucial complement proteins (e.g., C1q) in the blood (114, 115). Interestingly, CDC is increased when anti-cancer mAb therapy is combined with: (i) fresh-frozen plasma – a procedure in which previously frozen plasma containing complement proteins is infused into patients – to increase C1q bioavailability (116, 117); (ii) all-trans retinoic acid (ATRA) to modulate complement regulatory proteins (104, 118, 119); or (iii) complement regulatory protein blocking antibodies (120–122).

C1q is secreted by monocytes, macrophages, and immature dendritic cells *in vitro* (123–125). It has been shown that an individual bout of moderate to vigorous intensity exercise elicits a 100-480% increase in monocytes (55–57), and up to a 400% increase in immature dendritic cells (126). The mobilisation of monocytes and immature dendritic cells is transient, lasting approximately 20- to 30-minutes, and therefore, elevated C1q secretion is unlikely to occur prior to immune cell extravasation from the bloodstream. However, individual bouts of exercise that induces muscle damage (e.g., resistance training, ultra-endurance running) increases serum proteins of the C1-complex (e.g., C1s) for up to 3-days post-exercise (63–65) and further results in an influx of monocytes into damaged muscle, which differentiate into M2-like macrophages to facilitate the resolution of muscle injury (127–129). Thus, it might be hypothesised that increased intramuscular M2-like macrophage frequency in response to exercise-induced muscle damage results in increased C1q secretion that may ‘spill-over’ into the bloodstream 2- to 4-days post exercise (66, 67), improving mAb-mediated CDC.

Overcoming the restriction of CDC induced by complement regulatory proteins is a considerable challenge for mAb immunotherapy (21). Specifically, CD55 and CD59, regulate the complement classical pathway by accelerating the decay of C3/C5 convertases and preventing membrane attack complex formation, respectively (130–133). Current *in vitro* investigations to reduce complement regulatory proteins have focused on drugs (e.g., ATRA, anti-CD55, and anti-CD59 antibodies) (104, 118–122, 134) and cytokines (e.g., IFN-γ, TNF-α, IL-1α/β), and have primarily employed cell line models. However, the effects of cytokines are heterogenous and dependent on the cell line, cytokine, and complement regulatory protein investigated (135). The effects of individual exercise bouts on complement regulatory proteins warrants more research. Short (i.e., < 1 hour) and endurance style exercise (i.e., > 1 hour) of a moderate to vigorous intensity increases complement C3a (136, 137), which is indicative of elevated complement C3b. Increased complement C3b may overwhelm membrane bound complement regulatory proteins, a method previously suggested to augment mAb-mediated CDC (134).

The potential for exercise to improve mAb therapy through augmented CDC is an area yet to be explored. Studies investigating exercise and complement to date are heterogenous in their experimental and analytical methods and have tended to recruit healthy people (62). Thus, methodologically robust research is required to firstly characterise serum C1q kinetics and function following concentric and eccentric exercise among people with haematological cancers, and secondly to elucidate the effects of exercise-induced complement activation on complement regulatory protein expression.

### T-cell cytotoxicity

T-cell cytotoxicity (TCC) is induced by various anti-cancer therapies, including (i) bispecific antibodies (BsAbs) that combine the selectivity of a mAb with the therapeutic cytotoxic potential of T-cells (e.g., blinatumomab), and (ii) immune-checkpoint inhibitors for PD-1 (e.g., nivolumab), which block PD-1/PD-ligand(L)1 binding between T-cells and target cells to restore T-cell cytotoxicity (**Table 1**).

BsAbs are a form of immunotherapy for haematological cancers which have an anti-CD3 arm to engage CD3^+^ T-cells and a target antigen (e.g., anti-CD19, acute lymphoblastic leukaemia [ALL]; anti-CD269, myeloma; anti-CD20, non-Hodgkin’s lymphoma) arm to bind to target cells (27, 138, 139). The simultaneous binding of CD3 with a target antigen creates an immune synapse and induces perforin-mediated TCC via granzyme entry into malignant cells (**Figure 1F**) (140). Currently, blinatumomab – an anti-CD3/CD19 BsAb used to treat relapsed ALL (27) – is the only BsAb that is recommended by NICE (**Table 1**) and is therefore discussed herein. Blinatumomab activity is predominantly mediated by CD8^+^ effector memory T-cells (141–143), in a manner dependent upon the effector:target cell ratio (143). An individual bout of vigorous intensity cycling for 20-minutes increases the number of circulating effector memory T-cells by ~450% (44) and circulating CD19^+^ B-cells by ~100% (44) in healthy humans, and 30-minutes of vigorous intensity run-walk treadmill exercise increases total lymphocytes in blood by ~50% in children with ALL receiving maintenance therapy (144). Thus, the mobilisation of effector memory T-cells into blood may enhance blinatumomab responses by increasing the likelihood of blinatumomab-mediated formation of cytolytic synapses between CD19^+^ clonal B-cells and effector memory CD8^+^ T-cells. Furthermore, responders to blinatumomab therapy for ALL exhibit reduced T_regs_ compared to non-responders (145), and it has been shown that a single bout of treadmill walking/running at a moderate intensity can reduce circulating T_reg_ frequency people with CLL 1-hour after exercise (146). Although conflicting data has been reported for the effects of individual bouts of various modes of exercise on T_regs_ in a range of populations, with studies showing increases, decreases, or no change, as reviewed elsewhere (59). We note that three additional BsAbs: anti-CD3/CD269, teclistamab (138), and elranatamab (139); and anti-CD3/CD20, mosunetuzumab (147) are approved for the treatment of myeloma and non-Hodgkin’s lymphoma, respectively, by the US Food and Drug Administration (FDA), and are under review by NICE. The efficacy of these therapies may also be enhanced through an exercise-induced mobilisation of effector memory T-cells, thus improving TCC.

Blockade of PD-1 by mAbs has also been approved for the treatment of haematological cancers (**Table 1**). Chronic exposure of T-cells to tumour-associated antigens results in upregulated PD-1 expression which is indicative of T-cell exhaustion, and both PD-1 and PD-L1 overexpression on T-cells and target cells, respectively, is associated with worse survival (148–152) such as in myeloma (hazard ratio [HR] = 3.143), and diffuse large B-cell lymphoma (HR = 2.128) (149, 150). However, in the presence of anti-PD-1 therapy, an increased frequency of PD-L1^+^ cancer cells – which is associated with increased PD-1^+^ T-cells (153) – results in improved survival following anti-PD-1 therapy (154). A single bout of moderate to vigorous intensity cycling transiently increased the proportion of circulating PD-1^+^ T-cells (+3.2-5.3%) (45). In addition, mobilised T-cells expressed a greater density of PD-1 (+100-140%) than T-cells collected at rest (155), thus, increasing the binding potential between anti-PD-1 mAb and PD-1^+^ T-cells.

Another mechanism by which individual exercise bouts may enhance the efficacy of anti-PD-1 mAb is via increased frequency of stem-cell like memory T-cells (T_scm_); a self-renewing T-cell subset which provides persistent anti-tumour effector responses (156). Indeed, PD-1^+^ T_scm_ appear to mediate the restorative effects of anti-PD-1 therapy, as these cells proliferate to a greater extent than terminally-differentiated PD-1^+^ T-cells in response to anti-PD-1 therapy (157, 158). T_scm_ are induced by IL-7 and proliferate in response to IL-15 (159–161), which are myokines that may be secreted by skeletal muscle during moderate to vigorous exercise (162–164). Furthermore, T_scm_ share a naïve T-cell phenotype (CD45RA^+^CCR7^+^) (156), which increase by 42% following a single bout of vigorous cycling (46). Given that T_scm_ represent 2-3% of all circulating T-cells (156) it is plausible that T_scm_ represent a proportion (~7.5%) of the 42% increase to naïve T-cells in response to a single bout of exercise (46).

There are no studies which examine the effects of individual bouts of exercise on PD-1 therapy in haematological cancers. Investigations of exercise combined with PD-1 therapy in solid tumour models are mixed, showing beneficial, and no effects of exercise. For example, in a rodent model of breast cancer, synergistic benefits of treadmill running 5-days/week for 30-days combined with anti-PD-1 therapy and radiotherapy were shown when compared to anti-PD-1 therapy and radiotherapy without exercise training (165). In contrast, voluntary wheel running for 5-weeks before tumour cell inoculation, and then 2-weeks following tumour cell inoculation failed to augment anti-PD-1 therapy efficacy in a rodent model of melanoma (166). Conflicting findings in studies to date may be explained by differences in exercise and/or tumour models (26). The aforementioned studies rely on mobilised PD-1^+^ T-cells trafficking to tissues following exercise, and this may too be the case for B-cell lymphomas where the cancer cells exist in the lymphoid tissues. Previous research in a mouse model showed that following swimming, and running at 80% of $\dot{\text{V}}{\text{O}}_{2\max}$ until exhaustion, the frequency of labelled T-cells in primary, and secondary lymphoid tissues increases (167). Thus, it is plausible that T-cells mobilised into blood during an individual bout of exercise in humans, may migrate to lymphoid tissues following exercise cessation. On the other hand, for most haematological cancers, the migration of T-cells to tissues following exercise is not necessary given that the cancer cells exist predominately in the blood. Therefore, individual exercise bouts may improve the effectiveness of anti-PD-1 therapy both in blood, and in lymphoid tissue in haematological cancers.

In summary, the exercise-induced mobilisation of T-cells into blood is a well-known phenomenon that may augment both BsAb and anti-PD-1 mAb induced TCC against haematological cancers; yet it remains unknown whether T-cell mobilisation has adjuvant effects on immunotherapies that elicit their activity via TCC in haematological cancers. In addition, the ability of an individual exercise bout or long-term exercise training to modulate myokines (e.g., IL-7, IL-15) to promote the induction and proliferation of T-cell subsets (e.g., T_scm_) that elicit efficient TCC against haematological cancers requires further investigation.

### Clonal B-cell mobilisation

The efficacy of mAb therapies that elicit their effects via TCC, ADCC, ADCP, and CDC might be further enhanced by exercise bouts if target cells – along with effector cells/proteins – can be mobilised into the blood during treatment. Most haematological cancers are of B-cell lineage, and given that B-cells express β_2_-adrenergic receptors and are susceptible to an exercise-induced relocation into the blood (168, 169), it may be expected that B-cell lineage cancer cells may also be susceptible to exercise-induced redistribution.

In healthy people, CD19^+^ B-cell frequency in blood has been shown to increase by 100% in response 30-minutes of vigorous cycling (44). The most responsive B-cells possess an immature (CD20^+^CD27^−^CD38^+^) phenotype (+125%), followed by ‘B1’ cells (CD19^+^CD27^+^CD43^+^CD69^−^; +84%), memory (CD20^+^CD27^+^CD38^−^; +78%) and naïve (CD20^+^CD27^−^CD10^−^; +63%) B-cells (170). These exercise-induced B-cell responses are of interest given that the phenotype of human chronic lymphocytic leukaemia cells (CLL) – one of the most common blood cancers – is broadly consistent with the B1 cell phenotype (171), which appear highly responsive to bouts of exercise. Diffuse spread of CLL cells across lymphoid tissues can yield preferential survival of CLL clones in niches where external signals from the microenvironment promote their growth and survival (24), and where effector immune cells – such as NK-cells – appear to be limited in frequency (172). Additionally, rodent models of acute psychological stress – which induce a similar adrenergic response as exercise – appear to elicit a redirection of B-cells from the bone marrow (173), suggesting that at least some of the B-cells mobilised into blood during exercise may have lymphoid origins. These findings imply that exercise may be an effective adjunct – alongside other pharmaceutical methods such as Bruton’s Tyrosine Kinase (BTK) inhibitors (174, 175) – to relocate CLL cells from protective lymphoid niches into the blood, thus potentially bolstering mAb efficacy. Following mAb therapy, it might be hypothesised that exercise bouts could be used to assist in the detection of blood MRD in CLL, which is typically determined by flow cytometry (1, 171). For example, after treatment with conventional therapies, MRD persists at an ‘undetectable’ level in the blood of many patients, before CLL relapses and the disease is again detectable (15). It may therefore be the case that a bout of exercise could be used to mobilise CLL MRD from lymphoid tissues into blood, to provide early detection of relapsed disease. B-cells mobilised by exercise may also express CD79d and CD22 which are targets of palatuzumab vedotin (176) and inotuzumab ozogamicin (34), respectively. These mAbs are administered intravenously, and elicit their activity through a direct delivery of monomethyl auristatin E and calicheamicin following internalisation by the target cell, respectively, resulting in cell cycle arrest and apoptosis (34, 176). Thus, an individual bout of exercise may improve the direct delivery of cytotoxic agents by increasing the frequency of haematological cancer cells in blood, which could in turn facilitate the binding of mAbs to target cells when infused into the blood.

Unlike their B-cell lineage counterparts, plasma cells do not increase in blood during individual bouts of moderate to vigorous intensity exercise in healthy humans (170), but it is not known if exercise affects circulating plasma cell frequency in people with a greater plasma cell burden, such as people with myeloma. This warrants further research given that the mobilisation of clonal plasma cells may augment mAb-therapy, and could facilitate the detection of MRD in myeloma, for example, using the EuroFlow next-generation flow cytometry approach (177) as a non-invasive method to detect clonal B-cells in the peripheral blood of people with myeloma (178, 179), it was found that circulating myeloma cells – phenotyped as CD19^−^CD20^+^CD38^+^CD138^+^ – were present in 59% of people with myeloma pre-cursor, monoclonal gammopathy of undetermined significance, and 100% of people with smouldering multiple myeloma and multiple myeloma (178). Additionally, a greater burden of circulating myeloma cells was associated with a greater burden of myeloma cells in the bone marrow (178). It is also plausible that in the absence of plasma cell mobilisation, myeloma stem cells may be susceptible to exercise induced mobilisation as they resemble a memory B-cell phenotype (180), which can increase by 78% in healthy humans during moderate to vigorous aerobic exercise (170).

The complexity, and heterogeneity of haematological cancers makes their identification in blood during individual bouts of exercise a considerable challenge for future research. Indeed, some haematological cancer cells exist predominately in blood – such as CLL – and these cells can be identified by, for example, co-expression of CD5, CD19, and CD43, and clonality by kappa or lambda light chain restriction (1, 171). However, the identification of circulating cancer cells for other haematological cancers such as myeloma often requires a large volume of blood to be analysed (~5.1 mL) – due to the low frequency of circulating cells – and therefore large quantities of fluorochrome-conjugated antibodies. Further, the identification of circulating myeloma cells often requires a comparison between several proteins, requiring two antibody panels, and often the use of principal component analysis (177, 178, 181) – which attempts to distinguish normal from clonal myeloma cells based on their expression of several markers. Nevertheless, investigating new methods – such as exercise – to identify and treat MRD is a valuable area of future research given that persistent MRD results in relapse.

## Part 3: future perspectives

Lymphocytosis induced by individual bouts of exercise has primarily been described in healthy people (42–53, 55–58, 60, 61, 182), with limited evidence among patients with haematological cancers (144, 183). Evidence shows that an individual bout of exercise can induce profound changes to the blood immune cell compartment, including a transient increase in circulating NK-cells, monocytes, T-cells, and B-cells. This increase to circulating immune cell frequency could be harnessed to improve the depth of response during mAb therapy through augmentation of ADCC, ADCP, CDC, TCC, and direct delivery of cytotoxic agents. It is also hypothesised that an individual bout of moderate to vigorous intensity exercise may induce a relocation of clonal B-cells from peripheral tissues – and protective B-cell stromal niches – into blood, which in some cancers (e.g., CLL) may facilitate mAb therapy as both target and effector cells will be brought together with mAb in blood.

**Figure 2:**
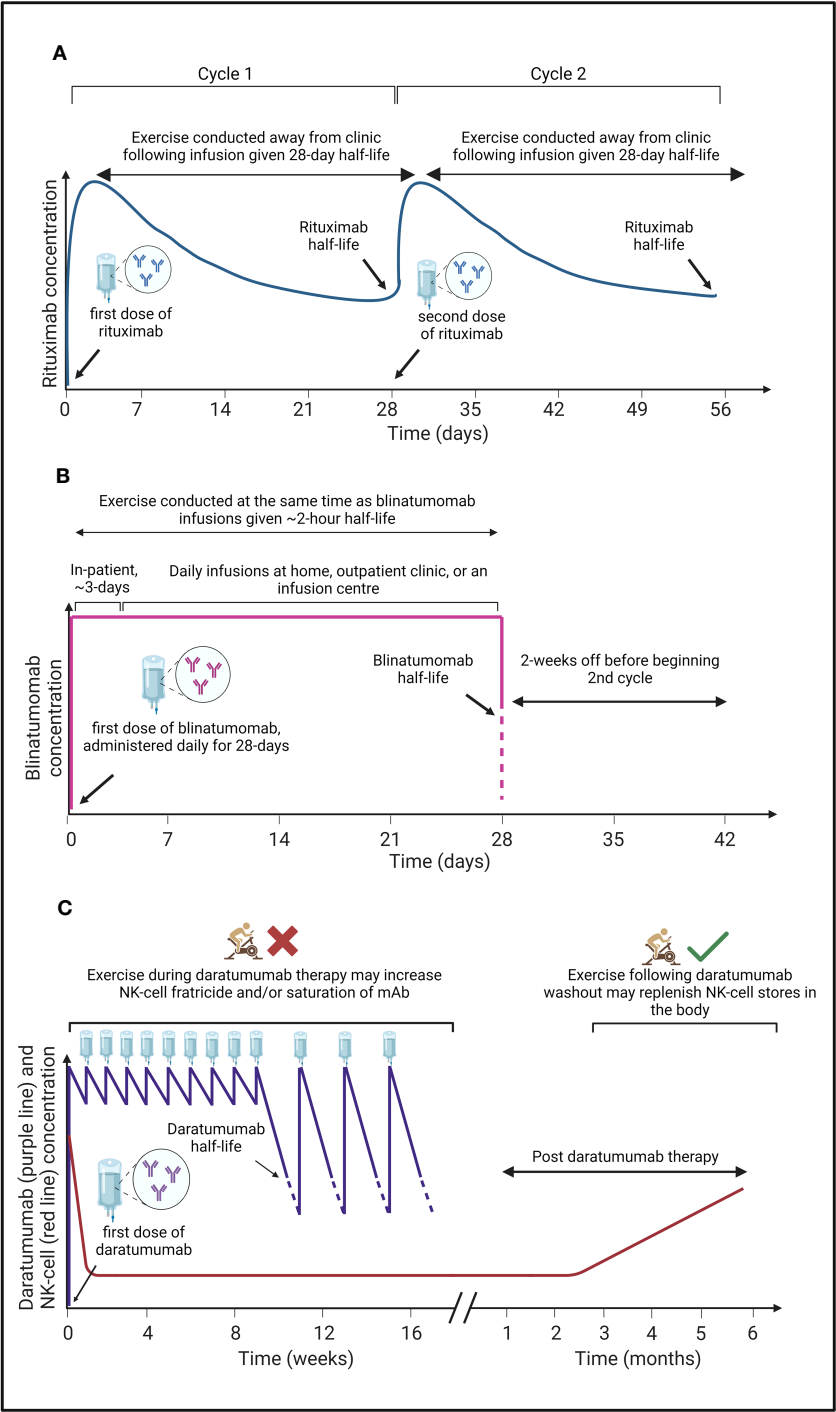
A graphical summary of the pharmacokinetics of three monoclonal antibodies (mAbs) used to treat haematological cancers, and a proposed ‘optimal period’ to undertake exercise. **(A)** Rituximab is administered in clinic with a half-life of ~28-days (184) at which time patients receive a second dose (cycle 2). Due to the relatively long half-life of rituximab, exercise can be conducted by patients outside of the clinic to induce a mobilisation of immune, and cancer cells into blood where rituximab is present, potentially bolstering mAb efficacy. **(B)** Blinatumomab has a short half-life of ~2-hours (185), therefore, patients are given daily infusions for 28-days with the first 3-days often administered in clinic and further doses administered at home, an outpatient clinic, or at an infusion centre before a 2-week break prior to a second cycle. To gain the most benefits from the acute exercise induced immunomodulatory effects, exercise should be conducted at the same time as blinatumomab infusions to mobilise immune cells and cancer cells into the blood where blinatumomab is present, potentially bolstering mAb efficacy. **(C)** Daratumumab has a half-life of ~9-days (186) and is administered weekly for the first 8-weeks, and then bi-weekly for a further 8-weeks before the time in-between doses increase. During treatment, NK-cell concentration decreases rapidly, before beginning to recover 3-months post daratumumab therapy (30), likely owing to NK-cell fratricide (76). Undertaking exercise at the same time as daratumumab infusions will mobilise NK-cells into the blood where daratumumab is present but where the frequency of myeloma cancer cells could be limited. Thus, exercise might be counterproductive by increasing NK-cell mediated fratricide and/or increase the saturation of daratumumab by NK-cells. The immunomodulatory effects of acute exercise may therefore be better harnessed following daratumumab washout when NK-cell concentration begins to recover.

To make use of exercise as an adjuvant therapy in combination with mAbs, the optimal timing, dose, and frequency of exercise must be considered, alongside the half-life and targets of different mAbs (**Figure 2**). Indeed, the half-life of mAbs range from 2-hours to more than 28-days. Thus, the optimal exercise prescription of exercise will need to be specific for each drug. For instance, daily intravenous infusion of BsAb-blinatumomab over 28-days – typically as an inpatient for the first ~3-days – is required due to its ~2-hour half-life (185, 187). Therefore, undertaking bouts of exercise *during* BsAb-blinatumomab infusion to repeatedly mobilise T-cells and clonal B-cells, might be optimal (**Figure 2B**). However, exercise *during* infusion might not be necessary for other drugs such as Rituximab given its half-life is relatively long (~28-days) after a single dose (184), and instead exercise could be performed away from the clinic (**Figure 2A**). Care must be taken with the timing of exercise during other mAb treatment. For example, exercise during daratumumab infusion may cause a rapid depletion of effector NK-cells – via a fratricide mechanism – due to the expression of target antigen (e.g., CD38) on the NK-cell surface (30, 76). Thus, undertaking an exercise bout prior to, and during daratumumab infusion could be counterproductive, and might result in increased NK-cell fratricide, or saturation of daratumumab by CD38^+^ NK cells rather than tumour cells. Instead, undertaking exercise following daratumumab washout may be favourable to restore the frequency of effector NK cells in the blood (**Figure 2C**). Additionally, participation in muscle damaging exercise during the infusion of mAb therapies which elicit their actions through CDC (e.g., rituximab) could be beneficial given that complement is increased up to 3-days following prolonged or muscle damaging exercise (63–65), which may comprise C1q (66, 67).

Lastly, upon considering a role for exercise as an adjuvant for haematological cancer mAb therapies, it is salient to consider the impact of age on the immune system, and thus, immunotherapy efficacy. Indeed, the majority of B-cell haematological cancers typically present in older individuals. For example, the median age at diagnosis for mature B-cell neoplasms is 71.8-years (188). As such, the immunomodulatory effects of individual bouts of exercise discussed herein may be influenced by the age associated decline in immune function – termed immunosenescence (189) – characterised by low frequencies and proportions of naïve T-cells, elevated frequencies of late-differentiated memory T-cells, and dysfunctional NK-cells (190, 191). Thus, in addition to the optimal timing, dose, and frequency of exercise, future research should consider participants age and its impact on immune competency.

## Conclusions

Therapeutic mAbs used for the treatment of B-cell haematological cancers is a compelling line of therapy in which to harness the immunomodulatory effects of individual exercise bouts. In this review, we summarised how individual exercise bouts might improve the efficacy of mAb therapy through augmentation of mAb modes of action including: ADCC, via mobilisation of NK-cells, and modulation of cell surface expression of inhibitory signals; ADCP, via mobilisation and redistribution of monocytes that may differentiate into haematological TAMs, and re-education of TAM phenotype from M2-like to M1-like that induce efficient ADCP in both circulation and protective stromal niches; CDC, via promoted extra-hepatic secretion of C1q by M2-like macrophages in damaged skeletal muscle; TCC, via mobilisation of T-cells, and modulating cell surface immune checkpoint receptors. Mobilisation of clonal B-cells in response to exercise bouts may further enhance these mechanisms in some haematological cancers (e.g., CLL, ALL), as well as improve the direct delivery of cytotoxic agents to target cells. Future research is required to demonstrate the feasibility and effectiveness of exercise as an adjuvant to mAb therapy. Indeed, studies need to examine the effects of both single and repeated bouts of exercise alongside mAb therapy, as well as determining exercise timing, duration, and frequency, and considering the half-life, target, and mechanisms of each mAb therapy.

## Author contributions

HC-B, FB, AC, AE, and JC contributed to the conception and design of the review. HC-B, FB, AC, and AE wrote sections of the manuscript. HC-B finalised the manuscript. All authors contributed to the article and approved the submitted version.
